# Ferroptosis-related lncRNAs as prognostic biomarkers in renal cell carcinoma: a systematic review and meta-analysis

**DOI:** 10.3389/fonc.2025.1579013

**Published:** 2025-05-23

**Authors:** Jia-Feng Lin, Zhi-Huang Wu, Min-Jie Zhang, Jian-Bin Luo, Guo-Qiang Chen

**Affiliations:** Department of Urology, The Second Hospital of Longyan, Longyan, Fujian, China

**Keywords:** renal cell carcinoma, ferroptosis, lncRNAs, prognosis, meta-analysis

## Abstract

**Background:**

Abundant evidences have indicated that long non-coding RNAs (lncRNAs) can be used to evaluate the prognosis of patients with renal cell carcinoma (RCC), and the purpose of this study was to evaluate the association between ferroptosis-related lncRNAs (FRLs) and the prognosis of patients with RCC by means of a meta-analysis.

**Materials and methods:**

All studies assessing the prognosis of patients with FRLs and RCC were collected up to 31 October 2024 by searching databases such as PubMed, Web of Science, Embase, Scopus and Cochrane Library. Pooled analyses were performed on the collected data, including metrics such as gender, age, risk score, tumor stage, and tumor grade. Hazard ratio (HR) and 95% confidence intervals (CI) were employed to assess the outcome metrics. To evaluate the heterogeneity among studies, the I² statistic and Q test were utilized. P < 0.05 was regarded as statistically significant. All data analyses were conducted by Stata 17.0 software and Review Manager 5.4.1.

**Results:**

19 literatures involving 5974 RCC patients were included in this study. The meta-analysis outcomes indicate that there was no significant correlation between FRLs and the gender of RCC patients (HR = 0.93, 95% CI = 0.85 - 1.03, P = 0.17). However, FRLs were associated with patient age (HR = 1.03, 95% CI = 1.03 - 1.04, P < 0.00001), risk score (HR = 1.05, 95% CI = 1.03 - 1.06, P < 0.00001), tumor grade (HR = 1.46, 95% CI = 1.28 - 1.67, P < 0.00001) and tumor stage (HR = 1.85, 95% CI = 1.68-2.03, P < 0.00001) were significantly correlated. In tumor staging, FRLs were significantly correlated with N-stage (HR = 1.51, 95% CI = 1.10 - 2.08, P = 0.01) and M-stage (HR = 1.80, 95% CI = 1.21 - 2.68, P = 0.004) in patients with RCC, but not significantly correlated with T-stage in patients (HR = 1.34, 95% CI = 0.86 - 2.09, P = 0.19).

**Conclusion:**

The findings of this study indicate that the abnormal expression of FRLs in RCC is obviously associated with the prognosis of patients, and that FRLs can be used as a new tumor marker to predict the prognosis of RCC patients with high accuracy.

**Systematic Review Registration:**

https://www.crd.york.ac.uk/PROSPERO/view/CRD42024610803, identifier CRD42024610803.

## 1 Introduction

Renal cell carcinoma (RCC), a neoplasm originating from renal epithelial cells, is primarily categorized into papillary RCC (pRCC) and clear cell RCC (ccRCC) (1, 2), and is the second most common and fatal urothelial tumor after bladder cancer, with an exponential increase in incidence in recent years (3). It is estimated that in 2020, approximately 430,000 new cases of RCC occurred, along with 180,000 deaths resulting from the disease (4). Despite the progress achieved in RCC diagnosis and treatment modalities in recent years, such as the improvement of surgical techniques and the application of immunotherapy and targeted therapy, the overall prognosis of RCC patients continues to be less than desirable, and the 5-year survival rate of patients with advanced RCC, in particular, is still low (5). Therefore, the search for effective prognostic biomarkers of RCC is of crucial importance for improving the clinical management of RCC patients, developing personalized treatment plans and increasing the survival rate of patients.

Ferroptosis, a novel form of regulated cell death identified in recent years, exhibits distinct characteristics that differentiate it from conventional cell death modalities, namely apoptosis and necrosis. Its underlying mechanism mainly involves iron metabolism disorders, lipid peroxidation and imbalance of the antioxidant system (6, 7). Ferroptosis has been implicated in the initiation, progression, and treatment resistance of numerous malignancies, including RCC (8). Long non-coding RNAs (lncRNAs) constitute a group of non-protein-coding RNA molecules with a length exceeding 200 nucleotides, which play a pivotal role in the regulation of gene expression, cell differentiation, development, and disease development (9, 10). Accumulating evidence suggests that lncRNAs can intricately regulate ferroptosis through diverse molecular mechanisms, such as directly interacting with ferroptosis-related proteins, regulating the expression of genes involved in iron metabolism, and influencing the level of lipid peroxidation (11).

In RCC, aberrant expression of ferroptosis-related lncRNAs (FRLs) is closely associated with tumorigenesis, progression, metastasis, and prognosis (12, 13). Some studies have found that specific FRLs are significantly over-expressed or under-expressed in RCC tissues and cells, and their expression levels are closely related to the clinicopathological features (e.g., tumor stage, grade, lymph node metastasis, etc.) and prognosis (e.g., overall survival, progression-free survival, etc.) of RCC patients (14–16). Collectively, these findings imply that FRLs hold promise as potential biomarkers for prognostic evaluation in RCC. They may also serve as novel targets and offer new directions for the implementation of precision medicine in the treatment of RCC, potentially enabling more tailored and effective therapeutic strategies.

However, no uniform conclusion has been drawn about the role of FRLs in the prognosis of RCC, and there are some differences and controversies among different studies. This may be related to a variety of factors, such as the sample size, study design, detection methods, and the types and functional complexity of lncRNAs. Therefore, it is necessary to conduct a systematic review and meta-analysis of existing relevant studies to comprehensively assess the value of FRLs as prognostic biomarkers in RCC, and to provide more reliable evidence for further clinical research and application. In this Meta-analysis, the standard procedure of meta-analysis will be strictly followed to comprehensively search multiple databases for studies on the relationship between the prognosis of RCC and FRLs, critically assess the quality of the included studies, and extract the relevant data for comprehensive analysis, to providing new ideas and methods for the assessment of the prognosis and clinical treatment of RCC.

## 2 Materials and methods

This meta-analysis was carried out in strict compliance with the Preferred Reporting Items for Systematic Reviews and Meta-Analyses (PRISMA) (17). The study protocol was registered on the PROSPERO website under the registration number CRD42024610803. The following operations were performed independently by both authors, in the event of any discrepancies or disagreements during these operations, a third author was consulted, and the issues were resolved through in-depth discussions.

### 2.1 Search strategy

The search strategy for this study used the medical subject terms of the search terms and their free terms up to 31 October 2024, and a total of studies from the following databases were searched (1) PubMed; (2) Embase; (3) Web of Science; (4) Cochrane Library; and (5) Scopus. And without any language restrictions. Taking PubMed as an example, the specific search strategy is as follows: [(long non-coding RNA) OR (RNA, Long Noncoding) OR (Long ncRNA) OR (lncRNA) OR (LincRNA) OR (LINC RNA)) AND (ferroptosis OR (iron death)] AND [(Kidney Neoplasms) OR (Kidney Neoplasm) OR (Renal Neoplasm) OR (renal cancer) OR (Renal Cancer) OR (renal carcinoma)]. The same search strategy was used for other databases. In addition, to avoid overlooking any potentially eligible studies, the references of the included studies were meticulously examined by hand.

### 2.2 Inclusion and exclusion criteria

The following were the inclusion criteria: (1) The study subjects were RCC patients and the expression levels of FRLs in RCC tissues were reported; (2) The included studies were required to provide relevant data on the relationship between the prognosis of RCC patients and the expression of lncRNAs, such as tumor stage, risk scores, and so on.

The exclusion criteria were defined as follows:(1) Studies that lacked relevance to the research topic or failed to furnish adequate data were excluded; (2) Studies with a sample size of less than 100 were not considered.; (3) Non-original research publications, including conference abstracts, case reports, and reviews were excluded.

### 2.3 Quality assessment

The quality of the included literature was evaluated using the Newcastle-Ottawa Quality Assessment Scale (NOS), which is a tool for assessing observational studies and is composed of three criteria based on study population selection criteria, comparability and outcome measures, where the study population selection criteria is out of a possible 4 points, whereas comparability and outcome measures are out of a possible 3 points out of a possible 9 points, with studies scoring ≥6 being considered as qualified studies.

### 2.4 Data extraction

Through careful reading of the full-text articles, the following crucial information was meticulously extracted, specifically study characteristics (the name of the first author, the year of publication, the country of publication, sample size), subject characteristics (age, gender), number of lncRNAs, and prognostic endpoints (tumor stage, risk scores, and tumor grading, etc.). To ensure accuracy, this process was progressed independently by two researchers, and any discrepancies that emerged during the extraction process were settled by consulting a third author.

### 2.5 Statistical analysis

In the present research, the association between RCC prognosis and FRLs was evaluated using hazard ratio (HR) and 95% confidence intervals (CI). To assess the heterogeneity across the incorporated studies, the I² statistic and Q test were employed. When the I² value was less than 50% and the P-value exceeded 0.05, a fixed-effects model was selected for analysis. Conversely, a random-effects model was utilized. Sensitivity analyses were conducted to appraise the reliability and stability of the results. Funnel plots were utilized to detect potential publication bias among the included studies. All statistical tests were two-sided and P < 0.05 was regarded as indicating a statistically significant difference. All statistical analyses were done by Stata version 17.0 (Statacorp, College Station, TX) and Review Manager Software version 5.4 (Cochrane, London, UK).

## 3 Results

### 3.1 Selection and characterization of studies

**Figure 1:**
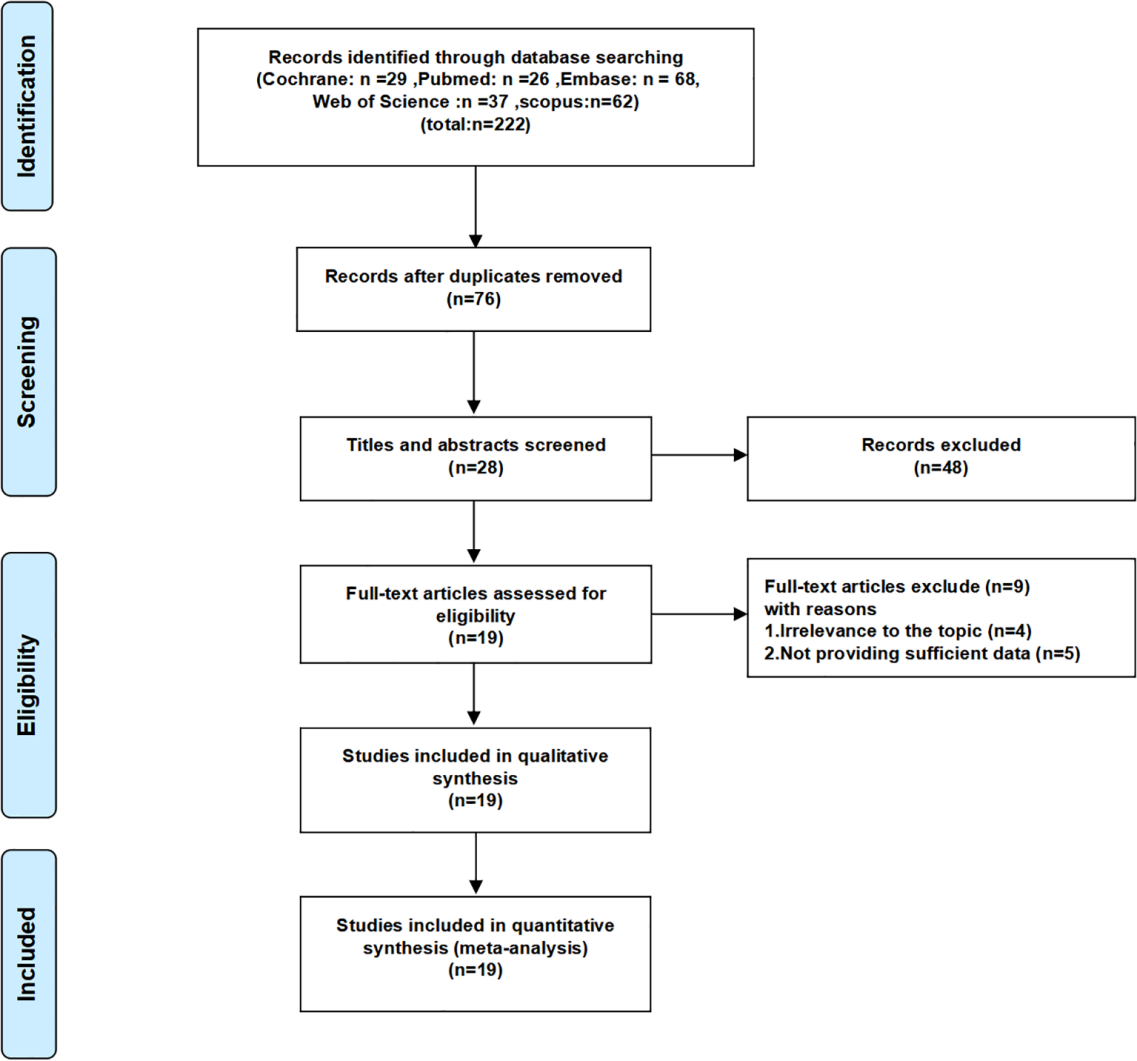
Flow chart of the screening process for the included studies.

222 studies were retrieved from the five databases mentioned above, and in the case of PubMed, the search formula is shown in **Supplementary Table S1**. Initially, 146 duplicate studies were removed. Subsequently, after perusing the titles and abstracts, 48 studies deemed irrelevant were excluded. Moreover, upon a meticulous review of the full text of 28 studies, 9 additional studies, which were either unrelated to the topic or failed to provide adequate data, were excluded. Ultimately, 19 studies were incorporated into this meta-analysis (13–15, 18–33). The screening procedure for the included studies is graphically depicted in **Figure 1**. A summary of the general characteristics of the included studies is presented in **Table 1**. All studies were recently published, and the vast majority were from China. The patients in the included studies were largely balanced at baseline. FRLs showed a strong predictive effect in the prediction of 1/3/5-year survival in RCC patients in most of the studies. **Supplementary Table S2** assesses the quality of the included studies. Given that all the scores of these studies were 6 or higher, it can be inferred that they met the required quality standards. **Supplementary Table S3** summarizes the FRLs screened in the included studies for predicting the prognosis of RCC patients.

**Table 1:** Characteristics of the 19 studies included in the meta-analysis.

Study	Country	Size	Sex (male/female)	TNM stage	Pathological type	1-year OS*	3-year OS*	5-year OS*	Refs
Zheng 2024	China	254	–	–	ccRCC	0.723	–	0.714	(13)
Zong 2023	China	265	178/87	129/31/56/47/2 (I/II/III/IV/NA)	ccRCC	0.765	0.745	0.805	(14)
Xiang 2023	China	470	313/157	230/47/193 (I-II/III-IV/NA)	ccRCC	0.779	0.756	0.788	(15)
Gong 2024	China	254	–	–	pRCC	–	0.913	0.808	(18)
Lai 2023	China	256	173/83	130/33/52/38/3 (I/II/III/IV/NA)	ccRCC	0.765	0.724	0.761	(19)
Ju 2022	China	262	176/86	154/108 (I-II/III-IV)	ccRCC	0.599	0.634	0.739	(20)
Wei 2022	China	149	104/45	77/14/29/29 (I/II/III/IV)	ccRCC	0.727	0.667	0.736	(21)
Liu 2022	Malaysia	149	100/49	89/60 (I-II/III-IV)	ccRCC	–	0.751	0.755	(22)
Han 2022	China	265	–	–	ccRCC	0.84	0.81	0.76	(23)
Zhu 2022	China	503	332/171	251/53/116/83 (I/II/III/IV)	ccRCC	0.76	0.66	0.71	(24)
Wu 2022	China	291	214/77	173/21/52/15/30 (I/II/III/IV/NA)	ccRCC	1	1	1	(25)
Dong 2022	China	269	–	135/28/57/49 (I/II/III/IV)	ccRCC	0.751	0.779	–	(26)
Zhou 2022	China	264	170/94	137/25/57/43/2 (I/II/III/IV/NA)	ccRCC	0.757	0.684	0.681	(27)
Chen 2022	China	530	344/186	322/205/3 (I-II/III-IV/NA)	ccRCC	0.763	0.735	0.766	(28)
Tang 2022	China	291	214/77	172/20/51/15/33 (I/II/III/IV/NA)	pRCC	0.908	0.884	0.821	(29)
Bai 2022	China	539	–	–	ccRCC	0.78	0.734	0.77	(30)
Shu 2022	China	409	276/133	216/40/88/50/15 (I/II/III/IV/NA)	ccRCC+ pRCC	–	–	–	(31)
Dang 2022	China	289	76/213	173/21/51/15/29 (I/II/III/IV/NA)	pRCC	0,930	0.953	0.933	(32)
Xing 2021	China	265	171/94	124/27/65/47/2 (I/II/III/IV/NA)	ccRCC	–	0.701	0.716	(33)

*AUC values for predicting 1-year/3-year/5-year overall survival in patients with renal cell carcinoma.

### 3.2 Meta-analysis results

**Figure 2:**
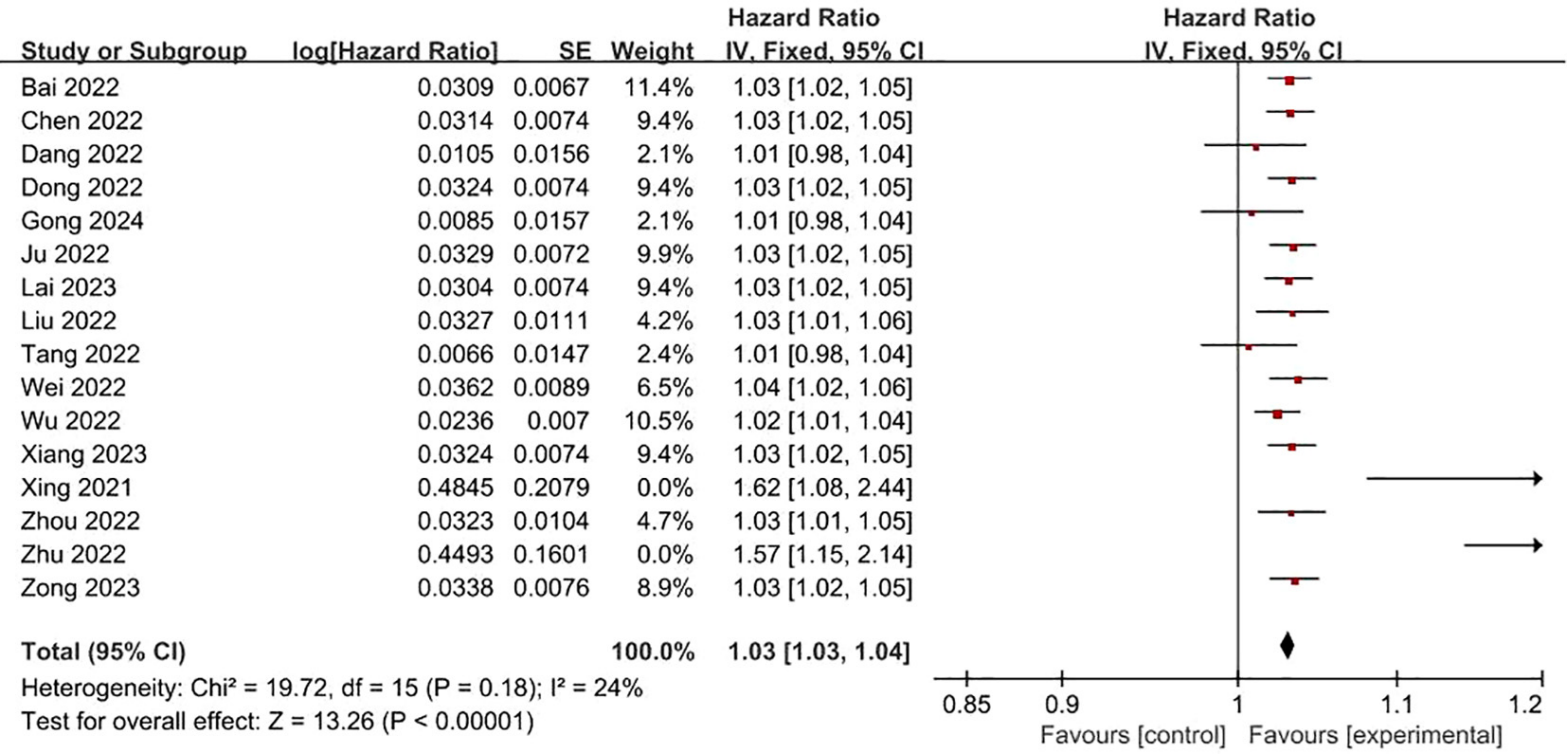
Forest plot of the relationship between FRLs and age.

**FRLs and age:** In total, 16 studies investigated and reported on the relationship between FRLs and patient age. Among these, 13 studies indicated a significant association between FRLs and patient age. Through meta-analysis, a significant correlation was further confirmed (HR = 1.03, 95% CI = 1.03 - 1.04, P < 0.00001). Notably, an assessment of heterogeneity among the included studies using the I^2^ statistic and P-value demonstrated no significant heterogeneity (I^2^ = 24%, P = 0.18). These correlation findings are visually presented in **Figure 2**.

**Figure 3:**
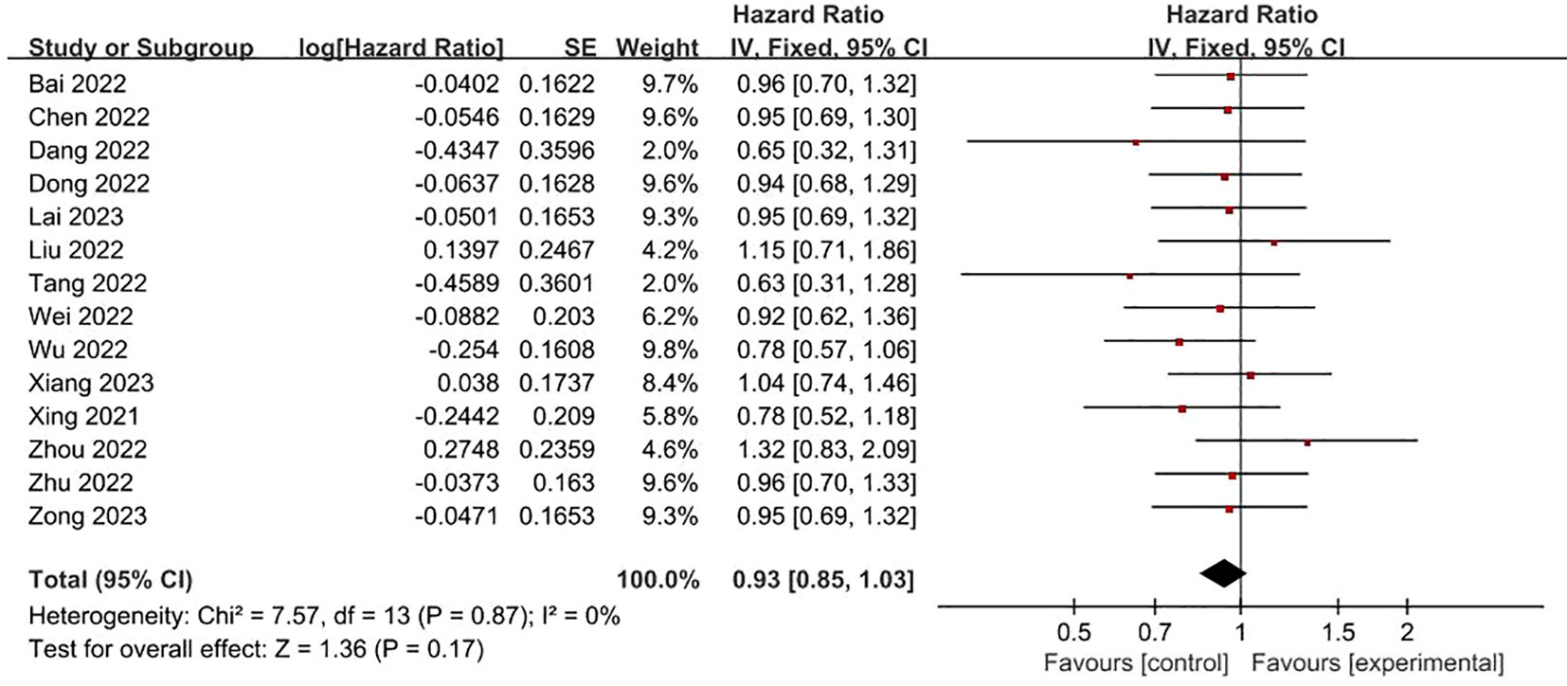
Forest plot of the relationship between FRLs and gender.

**FRLs and gender:** In all, 14 studies examined and reported on the relationship between FRLs and patient gender. Across these investigations, none of them found a significant correlation between FRLs and patient gender. Consistently, the meta-analysis outcome also indicated a lack of significant correlation between FRLs and patient gender (HR = 0.93, 95% CI = 0.85 - 1.03, P = 0.17). Moreover, an assessment of the heterogeneity among the included studies revealed that no significant heterogeneity among the included studies (I^2^ = 0%, P = 0.87). These findings are graphically represented in **Figure 3**.

**Figure 4:**
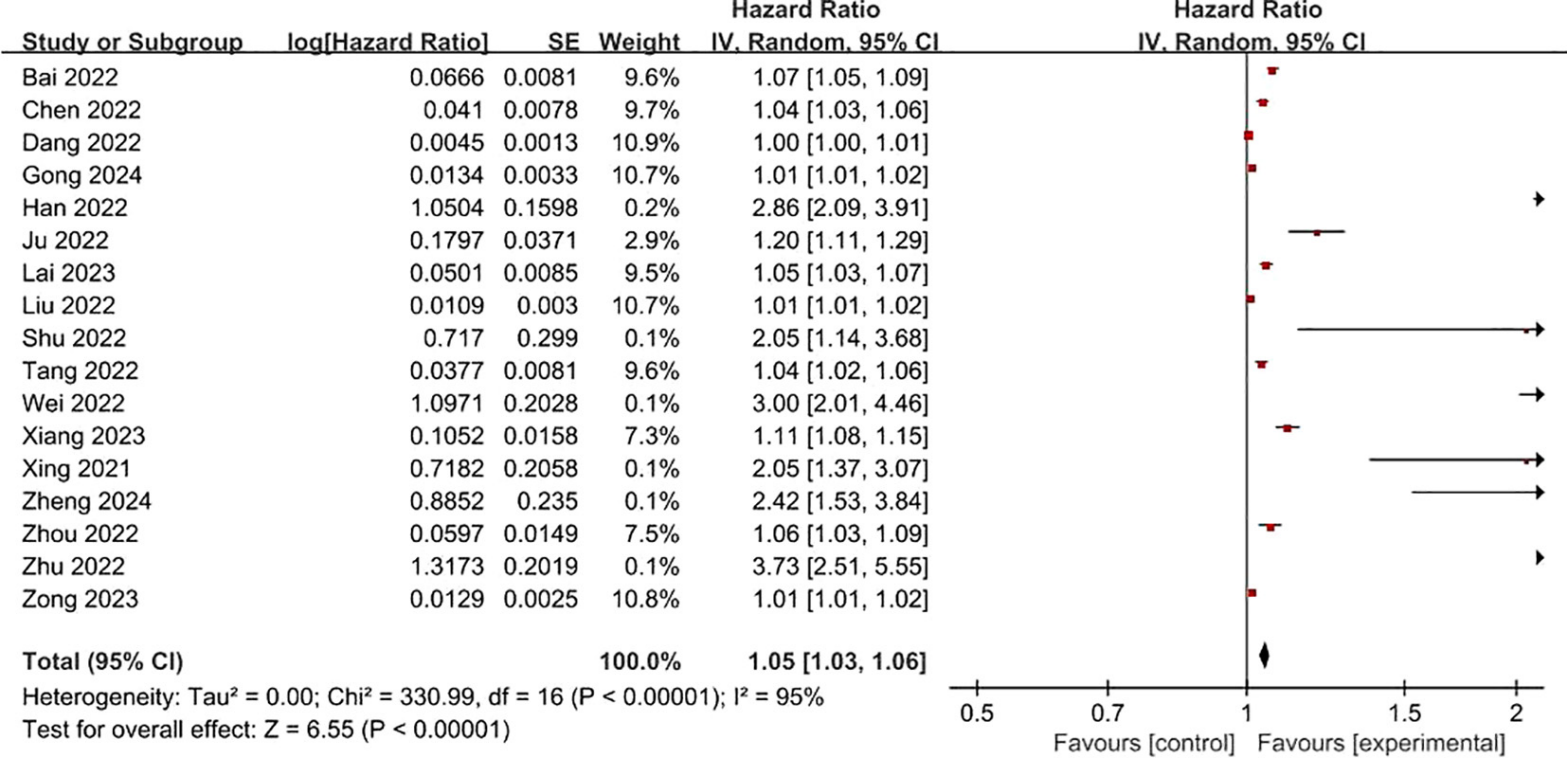
Forest plot of the relationship between FRLs and risk scores.

**Figure 5:**
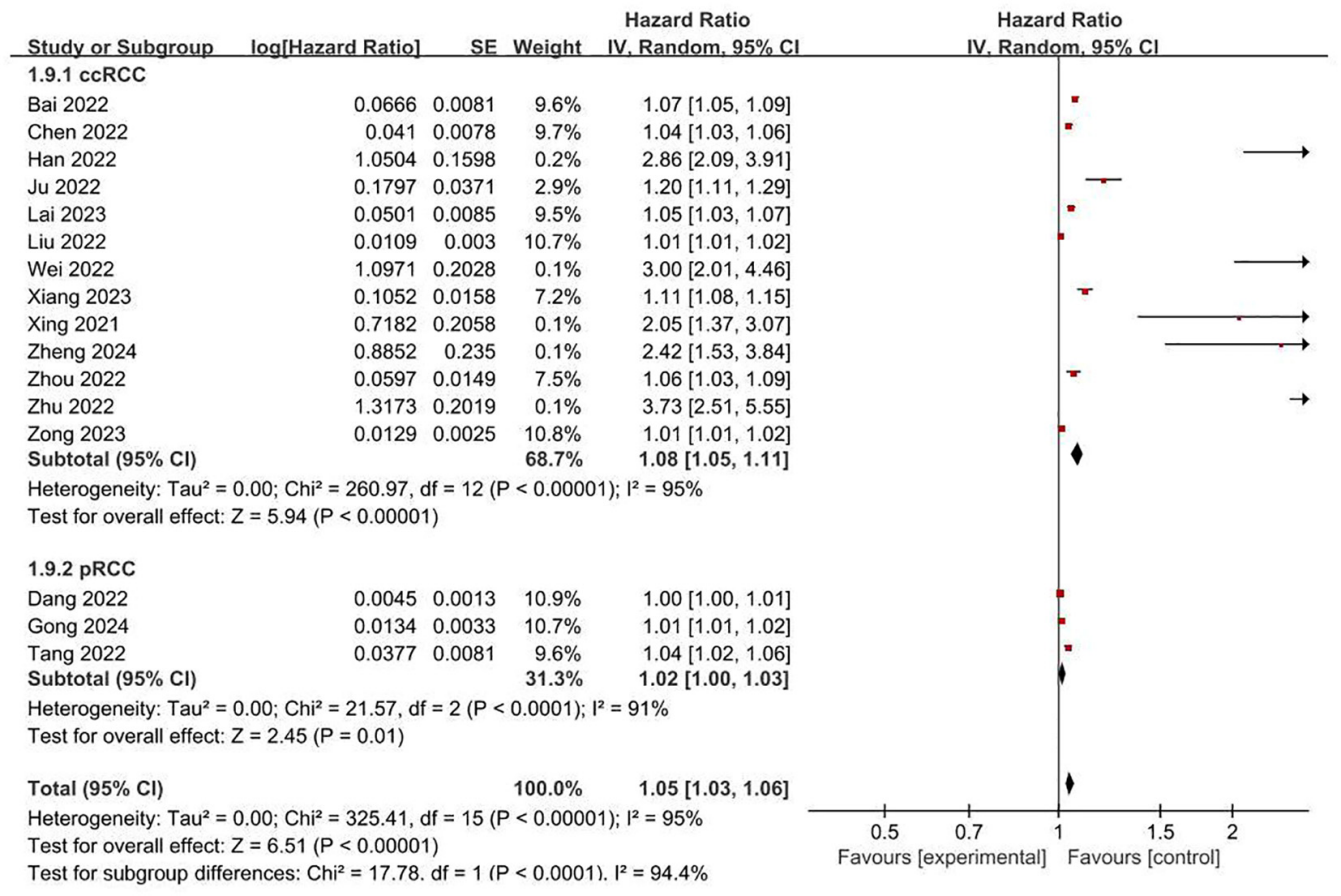
Forest plot of subgroup analyses on the correlation between FRLs and risk scores.

**FRLs and risk scores:** Regarding the association between FRLs and risk scores, 17 studies provided relevant reports. All of these 17 studies indicated a significant correlation between FRLs and risk scores. The outcome of the correlation meta-analysis further corroborated this significant relationship (HR = 1.05, 95% CI = 1.03 - 1.06, P < 0.00001). However, when assessing the heterogeneity among the included studies, a high degree of significant heterogeneity was detected (I² = 95%, P < 0.00001). These correlation findings are presented in **Figure 4**. Subgroup analyses based on RCC subtypes showed significant correlations between FRL and risk scores both in ccRCC and pRCC, but subgroup analyses did not reveal significant sources of heterogeneity. The correlation results are shown in **Figure 5**.

**Figure 6:**
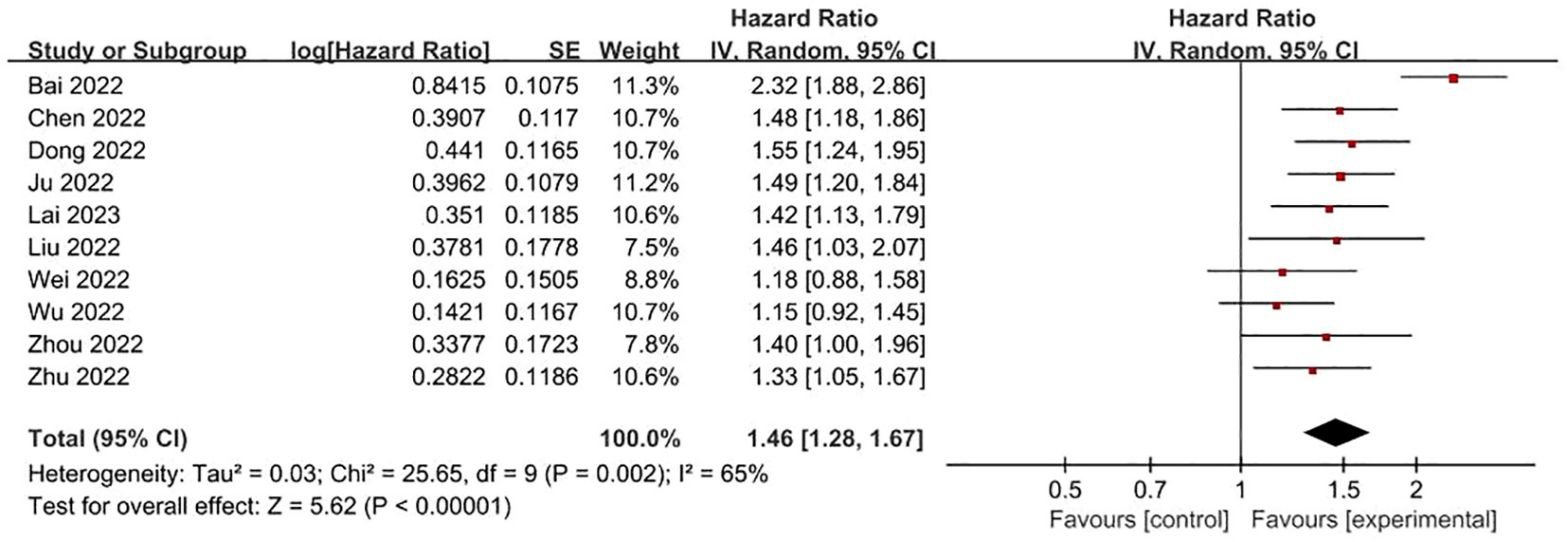
Forest plot of the relationship between FRLs and tumor grade.

**FRLs and tumor grade:** Concerning the relationship between FRLs and patients’ tumor grade, 10 studies furnished relevant reports. Among these, 7 studies demonstrated a significant correlation between FRLs and tumor grade. A corresponding meta-analysis was then conducted, and its result indicated a significant association between FRLs and tumor grade (HR = 1.46, 95% CI = 1.28 - 1.67, P < 0.00001). However, an assessment of the included studies revealed substantial heterogeneity (I² = 65%, P = 0.0002). The correlation outcomes are graphically presented in **Figure 6**.

**Figure 7:**
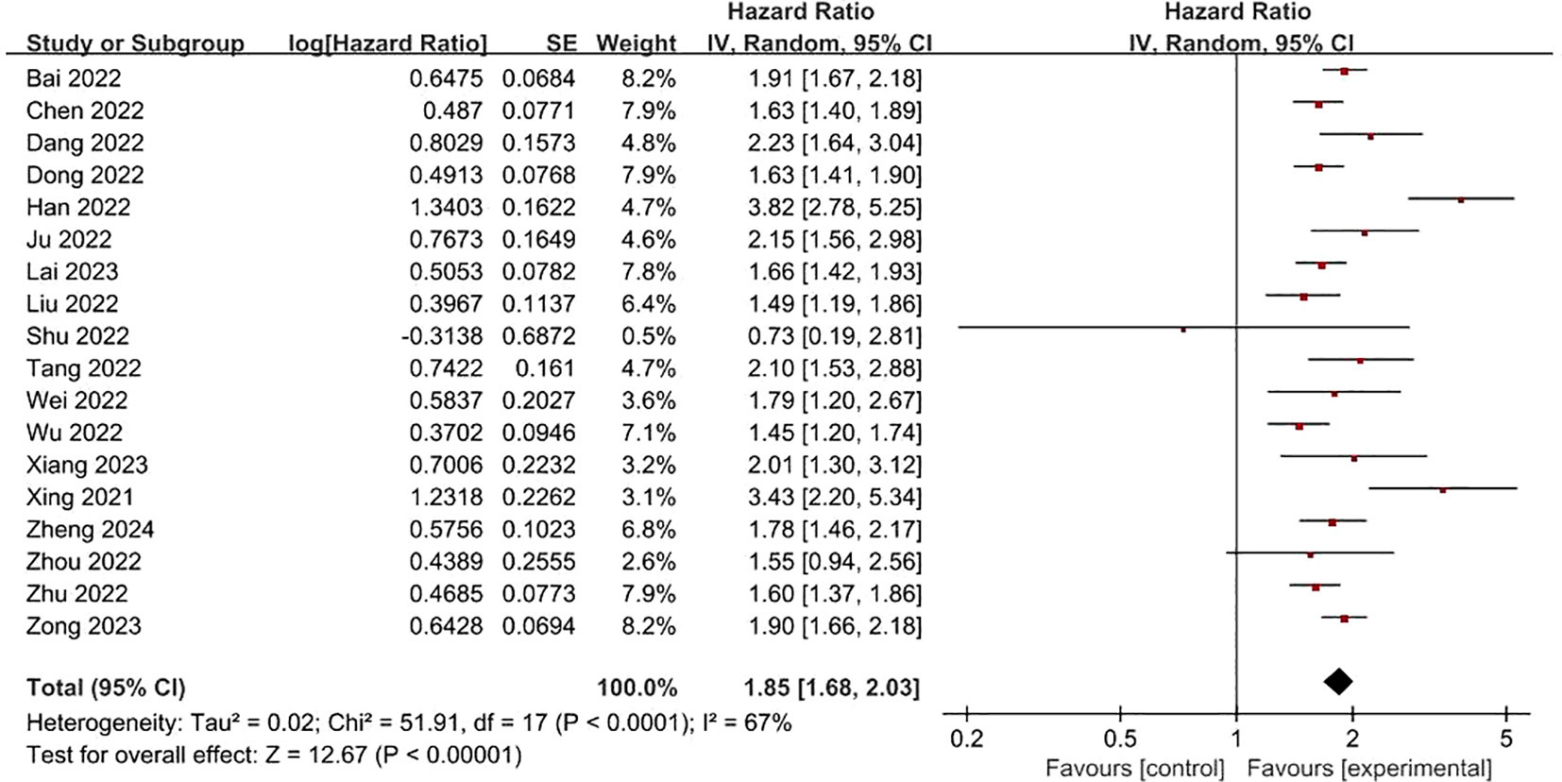
Forest plot of the relationship between FRLs and tumor stage.

**Figure 8:**
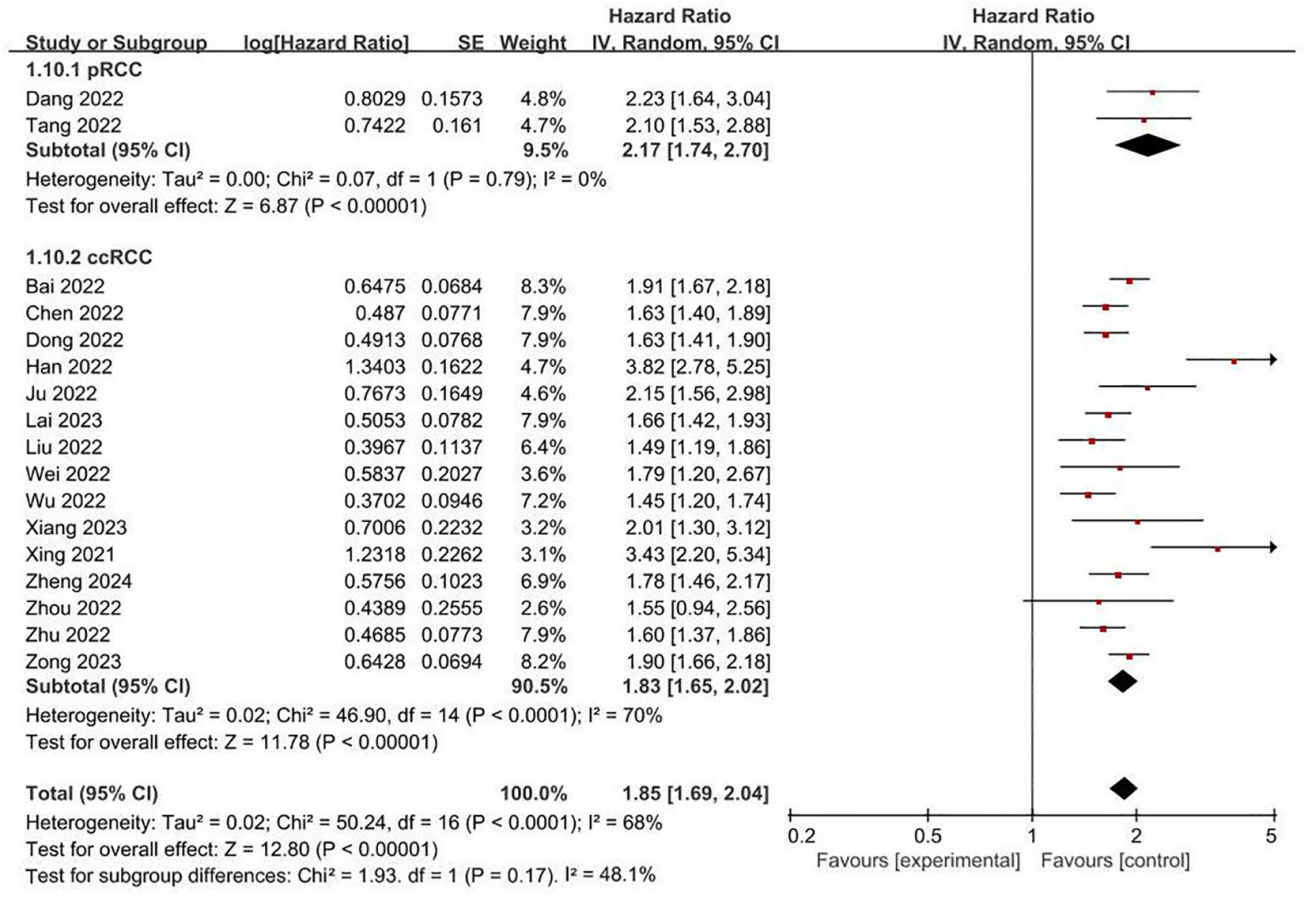
Forest plot of subgroup analyses on the correlation between FRLs and tumor stage.

**FRLs and tumor stage:** In addition, a total of 18 studies reported the relationship between FRLs and tumor stage in patients, of which 16 studies showed a significant correlation between FRLs and tumor stage, and meta-analysis result demonstrated a significant correlation between FRLs and tumor stage (HR = 1.85, 95% CI = 1.68-2.03, P < 0.00001), but significant heterogeneity existed among the included studies there was significant heterogeneity (I^2^ = 67%, P < 0.0001). The correlation results are shown in **Figure 7**. Subgroup analysis based on RCC subtypes revealed a significant correlation between FRL and tumor stage both in ccRCC and pRCC, but no significant heterogeneity was observed in the pRCC subtype, suggesting that the high heterogeneity may originate from the ccRCC subtype. The correlation results are shown in **Figure 8**.

**Figure 9:**
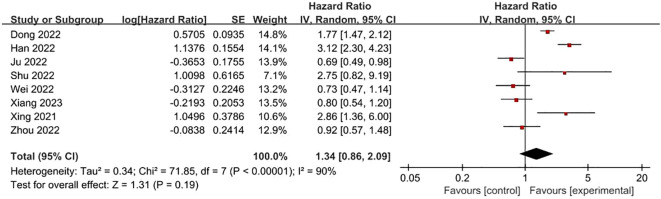
Forest plot of the relationship between FRLs and T-stage.

**FRLs and T-stage:** 8 studies reported the relationship between FRLs and patients’ T-stage, five of which showed no significant correlation between FRLs and T-stage, and similarly, the meta-analysis result demonstrated no significant correlation between FRLs and T-stage (HR = 1.34, 95% CI = 0.86-2.09, P = 0.19), however, there was significant heterogeneity among the included studies (I^2^ = 90%, P < 0.00001). The correlation results are shown in **Figure 9**.

**Figure 10:**
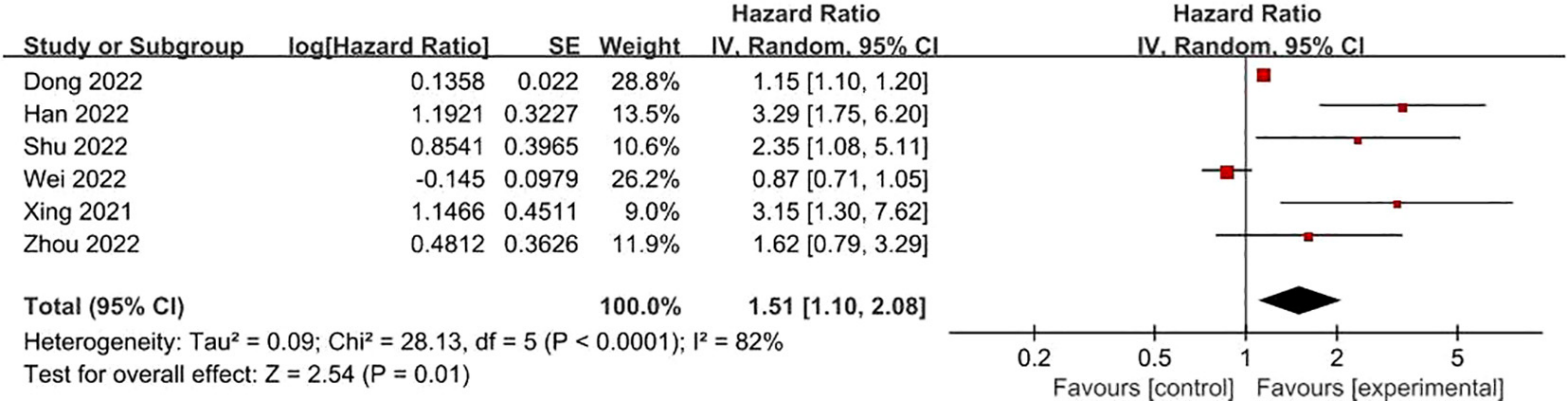
Forest plot of the relationship between FRLs and N-stage.

**FRLs and N-stage:** 6 studies reported on the relationship between FRLs and patients’ N-stage. Out of these, 4 studies indicated an obvious correlation between FRLs and the N-stage. Consistent with these individual findings, the meta-analysis result also demonstrated a significant correlation between FRLs and the N-stage (HR =1.51, 95% CI =1.10 - 2.08, P = 0.01). Nevertheless, a high level of significant heterogeneity was detected among the included studies (I² = 82%, P < 0.0001). The correlation results are depicted in **Figure 10**.

**Figure 11:**
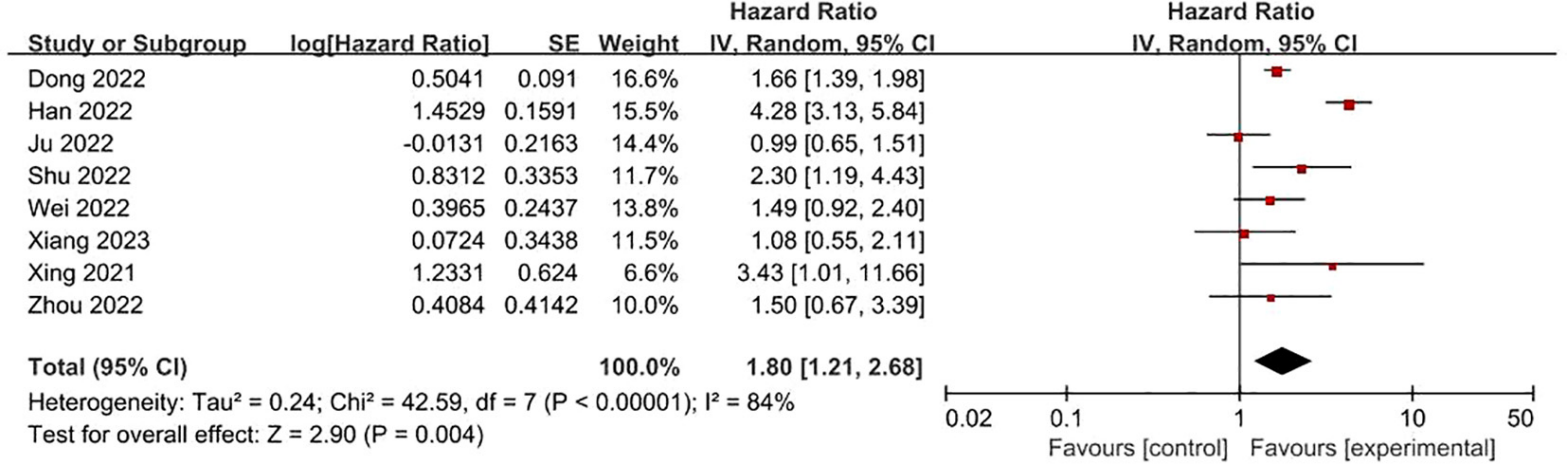
Forest plot of the relationship between FRLs and M-stage.

**FRLs and M-stage:** Similarly, eight studies presented reports on the relationship between FRLs and patients’ M-stage. Among these, four studies indicated a significant correlation between FRLs and the M-stage. In line with these individual study findings, the meta-analysis outcome also demonstrated a significant correlation between FRLs and the M-stage (HR = 1.80, 95% CI = 1.21 - 2.68, P = 0.004). Nevertheless, substantial heterogeneity was detected among the included studies (I² = 84%, P < 0.00001). The related results are illustrated in **Figure 11**.

### 3.3 Sensitivity analysis

**Figure 12:**
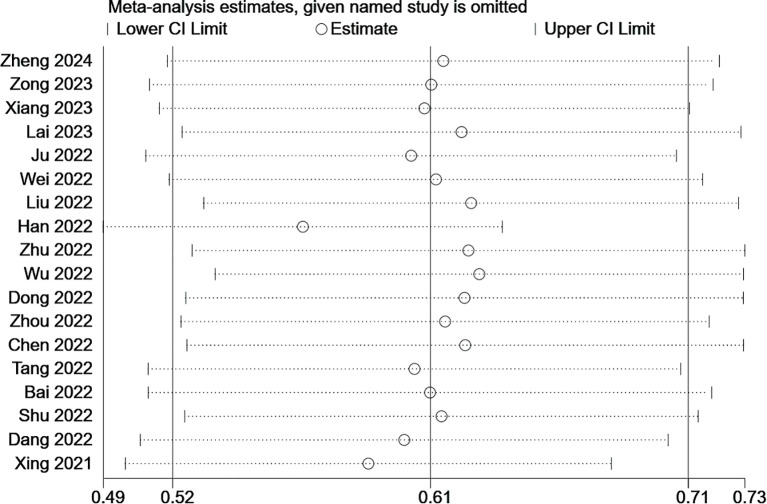
Sensitivity analysis based on tumor stage.

We assessed the reliability and robustness of the results by using sensitivity analyses. As the outcome of FRLs and tumor stage involved the largest number of studies, we performed a sensitivity analysis for this outcome. The outcome of the sensitivity analysis is shown in **Figure 12**, where any individual study had no significant effect on the results of the meta-analysis. Therefore, it is reasonable to believe that the results are relatively reliable.

### 3.4 Publication bias

**Figure 13:**
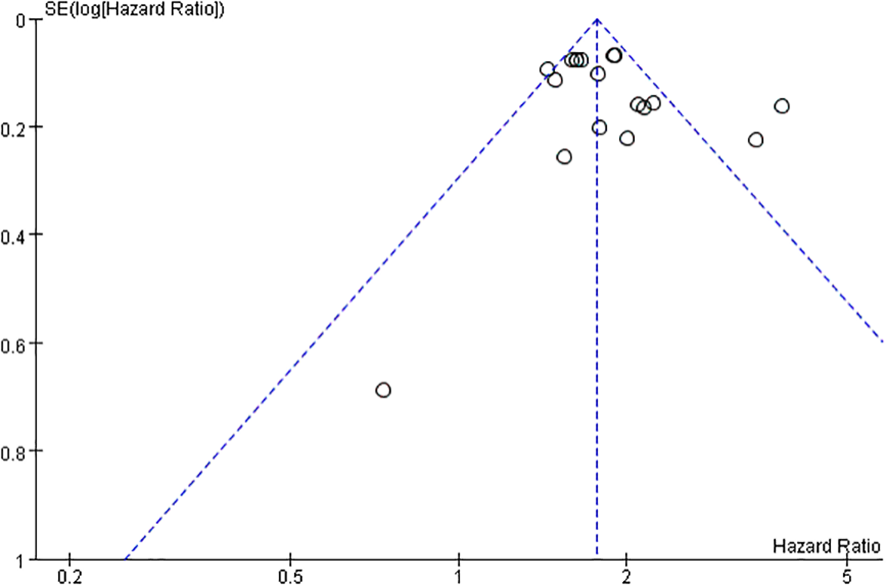
Funnel plot based on tumor stage.

**Figure 14:**
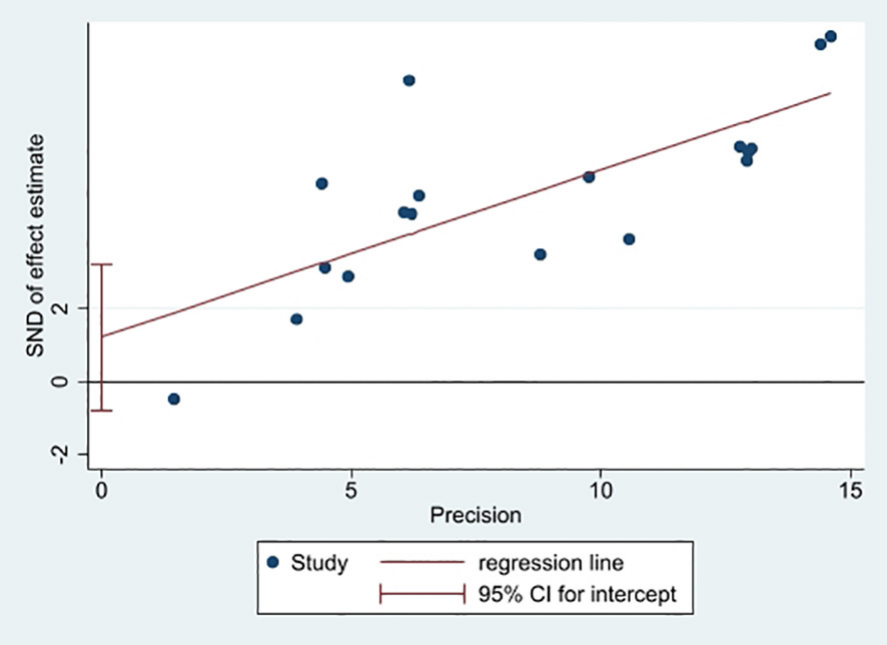
Plot of egger's test.

In this study, a funnel plot was utilized as a means to evaluate potential publication bias. Specifically, a funnel plot analysis was carried out for the relationship between FRLs and tumor stage. As depicted in **Figure 13**, the majority of the studies were positioned within the funnel plot, exhibiting good symmetry. This symmetry is typically an indication that there is no significant publication bias present. However, it was noted that three studies were situated outside the funnel plot. This deviation from the pattern of symmetry suggests that this meta-analysis might have been affected by some degree of publication bias. However, the egger test (P = 0.214) did not find significant publication bias, suggesting that our findings are relatively robust (**Figure 14**).

## 4 Discussion

RCC is a tumor with a very poor prognosis, with a strikingly low 5-year survival rate even after standardized anti-tumor therapy (34). To date, there is still a lack of efficient biomarkers to assess the prognosis of RCC. With the swift progress in transcriptomics technologies and molecular biology, lncRNAs have emerged as a novel focal point in cancer research (35). Ferroptosis has a profound connection to the development of RCC (16). In RCC, FRLs not only influence cancer cell proliferation, invasion and metastasis, but are also linked to patient prognosis (33). As potential prognostic markers of RCC, these lncRNAs hold promise in offering crucial references for the personalized treatment of RCC patients, helping doctors to predict the prognosis of patients more accurately, formulate more optimal therapeutic strategies, such as choosing the appropriate timing of surgery, postoperative adjuvant treatment options, and the application of immunotherapy. However, the predictive efficacy of FRLs in RCC prognosis remains to be confirmed.

In this study, we evaluated the predictive value of FRLs in RCC prognosis and revealed their potential clinical applications. The outcomes of this meta-analysis demonstrated that FRLs were significantly correlated with RCC prognosis. Despite the absence of significant disparities about gender and T-stage, the abnormal expression of FRLs was closely associated with age, tumor grade, tumor N-stage, M-stage, and risk score of RCC patients. These findings support the idea of FRLs as prognostic biomarkers in RCC. Metastasis is a pivotal determinant of poor tumor prognosis, with distant metastasis and lymph node metastasis being of great importance in tumor prognosis assessment (5, 36), and the expression levels of FRLs were significantly associated with distant metastasis and lymph node metastasis in RCC, and thus, they could be a potent predictor of RCC prognosis. Furthermore, it is worth noting that although the funnel plot based on tumor stage seemed to exhibit some publication bias, the egger test did not reveal significant publication bias, suggesting that the publication bias in this study was acceptable. The results of sensitivity analyses indicated the reliability of the results of this meta-analysis.

Although meta-analyses have shown that FRLs are effective predictors of RCC prognosis, how to achieve their clinical translation remains a major challenge. Current models for predicting prognosis, such as International Metastatic Renal Cell Carcinoma Database Consortium (IMDC)/Memorial Sloan Kettering Cancer Center (MSKCC), mainly rely on clinical parameters, and lack the ability to dynamically monitor molecular markers. In contrast, the use of FRLs for predicting the prognosis of RCC remains only at the molecular biology level. Using the expression level of FRLs (e.g., high-risk/low-risk grouping) as an additional variable, multivariate integration with the parameters of the IMDC/MSKCC clinical prognostic model, calculating their weight coefficients, and constructing a joint risk score will be beneficial to improving the predictive ability of RCC patients’ prognosis and identifying subgroups that have the same prognosis in the traditional model but with differences in the actual outcomes.

However, it is also important to recognize that there are some limitations to the findings of the study. Firstly, the overall sample size of the included studies was relatively small, and most of them are from China, which may have led to the results being less stable and representative. Although meta-analyses increase statistical efficacy to a certain extent, they may still prevent us from comprehensively and accurately assessing the prognostic value of FRLs across various RCC subtypes, different stages, and different treatment contexts. Second, significant heterogeneity was present among the studies. This manifested in multiple aspects such as differences in study design, patient population characteristics, lncRNAs detection methods, and data analysis method. Such heterogeneity can distort the accuracy and reliability of these results, making it difficult to accurately assess the prognostic value of certain lncRNAs. Although subgroup analyses are an important tool for addressing high heterogeneity, due to the limited data available from the original study, we performed subgroup analyses for only some of high heterogeneity outcomes based on RCC subtypes. Finally, a variety of lncRNAs associated with ferroptosis were included, and these lncRNAs have different expression statuses in RCC. However, due to the limited amount of data available in the literature, subgroup analyses of FRLs with different expression levels were not performed, and analyses of the prognostic impact of differently expressed lncRNAs on RCC were limited.

Given the limitations of this research and the current status of FRLs in the field of RCC research, future studies need to be improved and explored in depth in several aspects. Firstly, there is an urgent need for more large-scale and multicenter collaborative research endeavors. By increasing the sample size, the representativeness of the research findings can be significantly improved. These studies should be designed in strict accordance with standardized study protocols, including uniform patient inclusion and exclusion criteria, standardized clinical data collection methods, and standardized lncRNAs detection techniques and data analysis processes, to reduce inter-study heterogeneity and improve the reliability and comparability of study outcomes. Second, further in-depth exploration of the mechanistic roles of FRLs in RCC is warranted. Future studies could employ single-cell RNA-seq to resolve intratumoral heterogeneity or conduct functional studies to validate FRL-ferroptosis interactions, thereby investigating the mechanisms of FRLs at molecular, cellular, and tissue levels. This will provide a more robust theoretical foundation for their clinical translation. In addition, attention should also be paid to the interrelationships between FRLs and other known prognostic factors of RCC (e.g., tumor gene mutation status, immune microenvironment characteristics, etc.). Tumor development is a multifactorial, multistep, and complex process, and there may be synergistic or antagonistic effects between different prognostic factors. By comprehensively analyzing the combined effects of FRLs and other prognostic factors, it is expected to construct a more accurate and comprehensive prognostic prediction model for RCC, which will provide stronger support for clinical decision-making.

## 5 Conclusion

This meta-analysis initially explored the potential value of FRLs as a prognostic biomarker for RCC, and although there were some limitations and heterogeneity problems in the study, the outcomes of the meta-analysis indicated that FRLs had a significant correlation with RCC prognosis. In the future, more high-quality and multicenter collaborative research should be conducted to deeply investigate the mechanism of its action and comprehensively analyze it together with other prognostic factors, to further clarify the clinical application value of FRLs in the prognostic assessment of RCC, and to provide a new target and direction for the precise treatment of RCC.

## Data Availability

The original contributions presented in the study are included in the article/**Supplementary Material**. Further inquiries can be directed to the corresponding author.
