# Supplementary material for: Ferroptosis-related lncRNAs as prognostic biomarkers in renal cell carcinoma: a systematic review and meta-analysis
Source: Front Oncol. 2025 May 23;15:1579013. doi: 10.3389/fonc.2025.1579013 (PMC12141022; doi:10.3389/fonc.2025.1579013)
Supplement: Supplementary file 1 [file Table1.docx]

Table S1 The search formula of PubMed

| Search | Query | Results |
| --- | --- | --- |
| 1 | Ferroptosis | 14910 |
| 2 | Iron death | 11853 |
| 3 | #1 OR #2 | 22455 |
| 4 | RNA, Long Noncoding | 52267 |
| 5 | Long non-coding RNA | 55553 |
| 6 | Long ncRNA | 52397 |
| 7 | LncRNA | 59475 |
| 8 | LincRNA | 52488 |
| 9 | LINC RNA | 52372 |
| 10 | #4 OR #5 OR #6 OR #7 OR #8 OR #9 | 62848 |
| 11 | Kidney Neoplasms | 126340 |
| 12 | Kidney Neoplasm | 130108 |
| 13 | Renal Neoplasm | 133257 |
| 14 | Kidney Cancer | 157044 |
| 15 | Renal Cancer | 167438 |
| 16 | Renal carcinoma | 76334 |
| 17 | #11 OR #12 OR #13 OR #14 OR #15 OR #16 | 199976 |
| 18 | #3 AND #10 AND #17 | 26 |

Table S2 Quality assessment of included studies

| Study | Selection  (4 points) | Comparability  (2 points) | Outcome exposure measure  (3 points) | Total |
| --- | --- | --- | --- | --- |
| Zheng 2024^[1]^ | 3 | 2 | 3 | 8 |
| Zong 2023^[2]^ | 2 | 2 | 2 | 6 |
| Xiang 2023^[3]^ | 3 | 2 | 2 | 7 |
| Gong 2024^[4]^ | 3 | 2 | 2 | 7 |
| Lai 2023^[5]^ | 2 | 2 | 3 | 7 |
| Ju 2022^[6]^ | 3 | 2 | 3 | 8 |
| Wei 2022^[7]^ | 3 | 2 | 2 | 7 |
| Liu 2022^[8]^ | 3 | 1 | 2 | 6 |
| Han 2022^[9]^ | 2 | 2 | 3 | 7 |
| Zhu 2022^[10]^ | 3 | 2 | 2 | 7 |
| Wu 2022^[11]^ | 3 | 2 | 1 | 6 |
| Dong 2022^[12]^ | 3 | 2 | 2 | 7 |
| Zhou 2022^[13]^ | 3 | 2 | 3 | 8 |
| Chen 2022^[14]^ | 2 | 2 | 3 | 7 |
| Tang 2022^[15]^ | 3 | 2 | 2 | 7 |
| Bai 2022^[16]^ | 3 | 2 | 3 | 8 |
| Shu 2022^[17]^ | 3 | 2 | 2 | 7 |
| Dang 2022^[18]^ | 2 | 2 | 3 | 7 |
| Xing 2021^[19]^ | 2 | 2 | 2 | 6 |

Table S3 ferroptosis-related lncRNAs for predicting prognosis in renal cell carcinoma

| Study | Number | Name | Refs |
| --- | --- | --- | --- |
| Zheng 2024 | 6 | PVT1, CYTOR, MIAT, SNHG17, LINC00265 and LINC00894. | [1] |
| Zong 2023 | 8 | LINC00944, AP006545.1, ELDR, LINC01929, AC105429.1, PICSAR, AC124854.1 and LINC02348. | [2] |
| Xiang 2023 | 8 | LINC01615, AC026401.3, LINC00944, AL590094.1, DLGAP1-AS2, AC016773.1, AC147651.1 and AP000439.2. | [3] |
| Gong 2024 | 9 | SCN1A-AS1, MNX1-AS1, LINC01016, FAM230C, ZNF710-AS1, MIR100HG, SIRLNT, LINC01108 and LINC00896. | [4] |
| Lai 2023 | 8 | LINC00460, AC124854.1, AC084876.1, IGFL2-AS1, LINC00551, AC083967.1, AC073487.1 and LINC02446. | [5] |
| Ju 2022 | 7 | LINC00894, DUXAP8, LINC01426, PVT1, PELATON, LINC02609 and MYG1-AS1. | [6] |
| Wei 2022 | 7 | AC006129.2, CTB-41I6.2, CTD-2510F5.4, RP5-994D16.9, RP11-298J20.4, CTD-2396E7.11 and TUG1. | [7] |
| Liu 2022 | 17 | AC008742.1, AC010980.2, AC011700.1, AC084876.1, AC090337.1, AC139491.2, LINC01271, MANCR, PRKAR1B-AS1, TMEM246-AS1, AC004066.1, AC005722.3, AC007406.3, AC093583.1, AL928921.1, LINC02073, and PSORS1C3. | [8] |
| Han 2022 | 3 | AC124854.1, LINC02609 and ZNF503-AS2. | [9] |
| Zhu 2022 | 5 | LINC00460, LINC00894, VPS9D1-AS1, CYTOR, FOXD2-AS1. | [10] |
| Wu 2022 | 8 | CASC19, AC090197.1, AC099850.3, AL033397.2, LINC00462, B3GALT1-AS1, NCTAM34A and AC024022.1. | [11] |
| Dong 2022 | 23 | LINC00894, AL139123.1, AL157392.4, AL031714.1, LINC02804, KIF1C-AS1, AC114730.3, AC020907.4, AC088051.1, NALT1, AC245584.8, LINC00893, AC084876.1, AC012615.6, AC012645.4, AC003070.1, NFE4, MMP25-AS1, SLBP-DT, AC027796.4, AC005104.1, AC093788.1, and AC040162.3. | [12] |
| Zhou 2022 | 8 | AL590094.1, LINC00460, LINC00944, AC024060.1, HOXB-AS4, LINC01615, EPB41L4A-DT and LINC01550. | [13] |
| Chen 2022 | 7 | LINC00460, LINC00941, LINC02027, AC027271.1, AC026401.3, AC124854.1 and AC020907.4. | [14] |
| Tang 2022 | 5 | AC099850.3, LINC02535, LNCTAM34A, LINC00462 and FOXD2-AS1. | [15] |
| Bai 2022 | 9 | AC026401.3, LINC01615, PRKAR1B-AS1, LINC02609, LINC00460, AC084876.1, AC008870.2, LINC02747 and AC103706.1. | [16] |
| Shu 2022 | 5 | DOCK8-AS1, SNHG17, RUSC1-AS1, LINC02609 and LUCAT1. | [17] |
| Dang 2022 | 15 | ZFAS1, AC010624.2, AL031710.1, AL355102.4, MNX1-AS1, AC109460.1, AC127537.1, AC099850.4, LINC02154, AC024022.1, AC026401.3, LINC02535, ADAMTS9-AS1, AC107464.2, and MIR4435-2HG. | [18] |
| Xing 2021 | 3 | DUXAP8, LINC02609 and LUCAT1. | [19] |

**Reference**

1. Zheng, Q.; Gong, Z.; Lin, S.; Ou, D.; Lin, W.; Shen, P. Integrated analysis of a competing endogenous RNA network reveals a ferroptosis-related 6-lncRNA prognostic signature in clear cell renal cell carcinoma. *Advances in clinical and experimental medicine : official organ Wroclaw Medical University* **2024**, *33*, 1391-1407.

2. Zong, H.; Li, A.; Huang, Y.; Che, X.; Zhang, Y.; Ma, G.; Zhou, Z. Analysis of lncRNAs profiles associated with ferroptosis can predict prognosis and immune landscape and drug sensitivity in patients with clear cell renal cell carcinoma. *J Biochem Mol Toxicol* **2023**, *37*, e23464.

3. Xiang, X.; Guo, Y.; Chen, Z.; Zhang, F.; Qin, Y. Accurate prognostic prediction for patients with clear cell renal cell carcinoma using a ferroptosis-related long non-coding RNA risk model. *Cancer biomarkers : section A of Disease markers* **2023**, *37*, 95-107.

4. Gong, Y.; Zhang, C.; Li, H.; Yu, X.; Li, Y.; Liu, Z.; He, R. Ferroptosis-Related lncRNA to Predict the Clinical Outcomes and Molecular Characteristics of Kidney Renal Papillary Cell Carcinoma. *Current issues in molecular biology* **2024**, *46*, 1886-1903.

5. Lai, J.; Miao, S.; Ran, L. Ferroptosis-associated lncRNA prognostic signature predicts prognosis and immune response in clear cell renal cell carcinoma. *Scientific reports* **2023**, *13*, 2114.

6. Ju, L.; Shi, Y.; Liu, G. Identification and validation of a ferroptosis-related lncRNA signature to robustly predict the prognosis, immune microenvironment, and immunotherapy efficiency in patients with clear cell renal cell carcinoma. *PeerJ* **2022**, *10*, e14506.

7. Wei, S.Y.; Feng, B.; Bi, M.; Guo, H.Y.; Ning, S.W.; Cui, R. Construction of a ferroptosis-related signature based on seven lncRNAs for prognosis and immune landscape in clear cell renal cell carcinoma. *BMC medical genomics* **2022**, *15*, 263.

8. Liu, J.W.; Supandi, F.; Dhillon, S.K. Ferroptosis-Related Long Noncoding RNA Signature Predicts Prognosis of Clear Cell Renal Carcinoma. *Folia biologica* **2022**, *68*, 1-15.

9. Han, Z.; Wang, H.; Liu, Y.; Xing, X.L. Establishment of a prognostic ferroptosis- and immune-related long noncoding RNAs profile in kidney renal clear cell carcinoma. *Frontiers in genetics* **2022**, *13*, 915372.

10. Zhu, Z.; Zhang, C.; Qian, J.; Feng, N.; Zhu, W.; Wang, Y.; Gong, Y.; Li, X.; Lin, J.; Zhou, L. Construction and validation of a ferroptosis-related long noncoding RNA signature in clear cell renal cell carcinoma. *Cancer cell international* **2022**, *22*, 283.

11. Wu, Z.; Huang, X.; Cai, M.; Huang, P. Potential biomarkers for predicting the overall survival outcome of kidney renal papillary cell carcinoma: an analysis of ferroptosis-related LNCRNAs. *BMC urology* **2022**, *22*, 152.

12. Dong, Y.; Liu, D.; Zhou, H.; Gao, Y.; Nueraihemaiti, Y.; Xu, Y. A Prognostic Signature for Clear Cell Renal Cell Carcinoma Based on Ferroptosis-Related lncRNAs and Immune Checkpoints. *Frontiers in genetics* **2022**, *13*, 912190.

13. Zhou, Z.; Yang, Z.; Cui, Y.; Lu, S.; Huang, Y.; Che, X.; Yang, L.; Zhang, Y. Identification and Validation of a Ferroptosis-Related Long Non-Coding RNA (FRlncRNA) Signature to Predict Survival Outcomes and the Immune Microenvironment in Patients With Clear Cell Renal Cell Carcinoma. *Frontiers in genetics* **2022**, *13*, 787884.

14. Chen, X.; Tu, J.; Ma, L.; Huang, Y.; Yang, C.; Yuan, X. Analysis of Ferroptosis-Related LncRNAs Signatures Associated with Tumor Immune Infiltration and Experimental Validation in Clear Cell Renal Cell Carcinoma. *International journal of general medicine* **2022**, *15*, 3215-3235.

15. Tang, X.; Jiang, F.; Wang, X.; Xia, Y.; Mao, Y.; Chen, Y. Identification of the Ferroptosis-Related Long Non-Coding RNAs Signature to Improve the Prognosis Prediction in Papillary Renal Cell Carcinoma. *Frontiers in surgery* **2022**, *9*, 741726.

16. Bai, Z.; Zhao, Y.; Yang, X.; Wang, L.; Yin, X.; Chen, Y.; Lu, J. A Novel Prognostic Ferroptosis-Related Long Noncoding RNA Signature in Clear Cell Renal Cell Carcinoma. *Journal of oncology* **2022**, *2022*, 6304824.

17. Shu, X.; Zhang, Z.; Yao, Z.Y.; Xing, X.L. Identification of Five Ferroptosis-Related LncRNAs as Novel Prognosis and Diagnosis Signatures for Renal Cancer. *Frontiers in molecular biosciences* **2021**, *8*, 763697.

18. Dang, R.; Jin, M.; Nan, J.; Jiang, X.; He, Z.; Su, F.; Li, D. A Novel Ferroptosis-Related lncRNA Signature for Prognosis Prediction in Patients with Papillary Renal Cell Carcinoma. *International journal of general medicine* **2022**, *15*, 207-222.

19. Xing, X.L.; Yao, Z.Y.; Ou, J.; Xing, C.; Li, F. Development and validation of ferroptosis-related lncRNAs prognosis signatures in kidney renal clear cell carcinoma. *Cancer cell international* **2021**, *21*, 591.
